# Successful Outcome of Severe Intra-cerebral Bleeding Associated with Acquired Factor V Inhibition: Utilization of Multiple Therapeutic Agents

**DOI:** 10.4274/balkanmedj.2017.0158

**Published:** 2018-01-20

**Authors:** Panagiotis Andreadis, Katerina Kafantar, Aleka Agapidou, Sofia Vakalopoulou, Efthymia Vlachaki

**Affiliations:** 1 Clinic of Internal Medicine, Ippokrateio General Hospital of Thessaloniki, Thessaloniki, Greece; 2 Clinic of Hematology, Homerton University Hospital, Homerton Row, London

**Keywords:** Haemophilia, factor V, rituximab, inhibitor, cephalosporin

## Abstract

**Background:**

Acquired coagulation factor inhibitors are antibodies that either inhibit activity or increase the clearance of a clotting factor and lead to an increased risk of bleeding. Most of the time, the disorder is attributed to factor VIII inhibition (acquired haemophilia A); however, other coagulation factors could also be implicated.

**Case Report:**

Herein, we report an interesting case of a patient who underwent coronary artery bypass grafting and received antibiotic treatment after surgery with third generation cephalosporin. A month later, he presented with extreme bleeding diathesis and cerebral haemorrhage. Following a thorough clinical and laboratory investigation, an acquired factor V inhibitor was diagnosed. The patient received treatment with corticosteroids, intravenous immunoglobulins, anti-CD20 monoclonal antibodies (rituximab), cyclophosphamide and recombinant factor VIIa. Finally, despite the poor initial prognosis, the patient managed to achieve a full recovery.

**Conclusion:**

As there are no clear guidelines on acquired coagulation inhibitor treatment, reports of such cases could offer insight for future therapy choices. The case was unique because the treatment regimen included a combination of multiple therapeutic agents including rituximab.

Acquired inhibitors of coagulation is a rare disorder (1,2). Patients with no previous history of bleeding predisposition present with mild to severe bleeding diathesis (1,3). There are challenges in diagnosing acquired coagulation inhibition (2,4). Most of the time the disorder can be attributed to a factor VIII inhibitor (acquired haemophilia A-AHA) (5), but inhibition of other factors is also a possibility. Emergence of inhibitors has been correlated with multiple factors (6). Factor V inhibitor reports were common amongst surgical patients that were exposed to bovine thrombin during surgery (7) but other causes have been reported (8).

## CASE PRESENTATION

**Figure 1:**
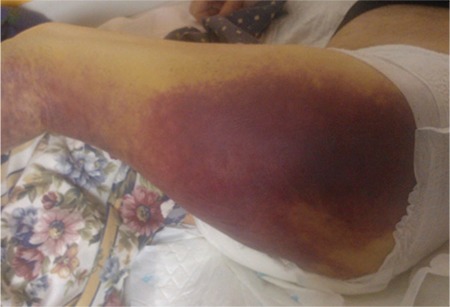
Extensive ecchymosis on the patient's left thigh.

A 78-year-old male presented to the emergency department due to repeated episodes of syncope over the last 3 days. Physical examination during admission revealed pallour and extensive ecchymosis in his left hemithorax and left thigh (Figure 1). The rest of the clinical and neurological examination revealed no pathological findings.

A month before admission, he underwent double coronary artery bypass grafting (CABG) due to coronary artery disease (CAD). His medications after CABG were acetylsalicylic acid (ASA) 100 mg, simvastatin 40 mg and ezetimibe 10 mg. Due to the extent of the surgical trauma, he also received antibiotic therapy with ceftazidime for a week. Two weeks later, the patient developed gingival bleeding. ASA was stopped and he received treatment with low molecular weight heparin (LMWH) (tinzaparin 14000 IU). He had no history of liver disease, bleeding disorders or any previous blood product transfusion.

He underwent a brain computed tomography (CT) scan that revealed a small intracerebral bleeding site on the left frontal lobe and a smaller bleeding site in his right occipital horn. Laboratory results were noteworthy. Haematocrit (Ht) was 23% and haemoglobin was 7.4 g/dL and platelet (PLT) count was 140x103/μL. Extreme prolongation of prothrombin time (PT), international normalized ratio (INR) and partial thromboplastin time (APTT) were noticed in the coagulation screen. (PT: 62.9 s, INR: 5.71, APTT: above upper measurable limits) (Table 1). The rest of the lab results were within normal ranges.

**Figure 2:**
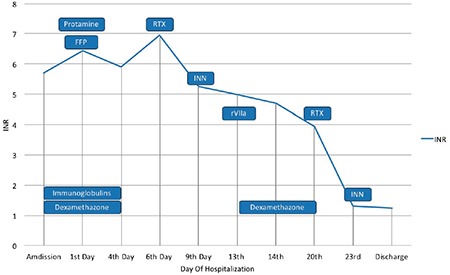
Diagram depicting international normalized ratio flunctuation and administered treatment. Patient received IV immunoglobulins and dexamethazone from admission till 4^th^ day of hospitalization. On the 1^st^ day he was transfused with FFP and protamine sulfate and Vit K was also administered. On Day 6 RTX was administered followed by cyclophosphamide (INN) during 9^th^ day of stay. Patient received treatment with rVIIa on the 13^th^ day due to deterioration of clinical condition due to intracerebral hemorrhage. Rituximab and INN infusions were administered on day 20^th^ and 23^rd^ respectively. *FFP: fresh frozen plasma; IVIG: IV immunoglobulins; RTX: rituximab; rVIIa: recombinant Factor VIIa; INR: international normalized ratio*

The patient was transfused with 3 red blood cells units and 6 fresh frozen plasma (FFP) units (vitamin K was infused before transfusion). The Ht value was stable after the transfusion (Ht >30%) but PT and APTT were still prolonged (Figure 2). Due to the recent history of LMWH treatment, protamine sulfate was also administered but with no results.

The irreversibility of the patient’s clotting assays through exogenous clotting factor transfusion and LMWH antidote (FFP and protamine sulfate accordingly), and the inability to further correct coagulation test abnormalities with a 50:50 mixing test lead to the suspicion of an acquired coagulation factor inhibitor. Immunologic and viral screening, protein electrophoresis and lupus anticoagulation tests were negative. Haemophilia test results revealed the presence an inhibitor of factor V [64 Bethesda units (BU)]. The inhibition was so potent that the levels of all factors in the common coagulation cascade pathway were also influenced (Table 1).

**Figure 3:**
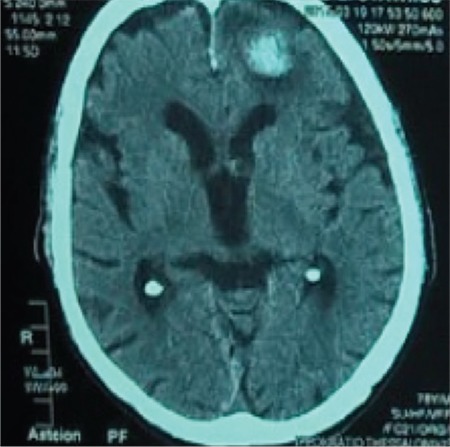
Brain computed tomography revealing a large intracerebral hemorrhagic site on the left temporal lobe and perifocal edema.

Treatment with intravenous steroids, daily dexamethasone 40 mg, and immunoglobulins (400 mg/kg) was started for 4 consecutive days but clotting assays showed no signs of improvement (Figure 3). During day 6 post-admission, he received treatment with anti-CD20 monoclonal antibody, rituximab (Rituxan, MabThera, Genetech-300 mg) and methylprednisolone 16 mg twice daily. On day 9, cyclophosphamide (700 mg) was also administered, again without a significant improvement of the clotting screen. The patient was in relatively good clinical condition with no signs of new ecchymosis and no neurologic deficit.

Following cyclophosphamide administration, the patient presented a low grade fever (37.5 °C) even though white blood cell count remained normal (6000/μL). Due to repeated episodes of fever and the patient’s complaints regarding dysuria, blood and urine cultures were obtained and treatment with levofloxacin was started due to a possible urinary tract infection.

Four days later (day 13), the patient presented with focal seizures of the right upper extremity that were generalised and had a left gaze shift. During the postictal state, he was lethargic with dysarthria. After the administration of clonazepam and levetiracetam, the patient was stable and a new brain CT scan revealed a large intracerebral haemorrhagic site on the left temporal lobe with perifocal oedema and multiple smaller bleeding sites (Figure 3). Levofloxacin was stopped since quinolones such as levofloxacin are implicated in seizures. Antibiotic treatment was changed to piperacillin/tazobactam due to positive urine cultures for Pseudomonas aeruginosa despite the correlation between antibiotics and coagulation inhibitors. The patient received treatment with dexamethasone and recombinant human coagulation factor VIIa (rFVIIa) (80 mcg/kg bolus every 3 hours) for 48 hours. PT, APTT and INR slowly decreased (52.7 s, 112.3 s and 4.77 s respectively). Rituximab and cyclophosphamide infusions were repeated during the 20th and 23rd day of hospitalisation. INR showed a significant drop from 3.94 to 1.31 and remained stable for the remainder of the hospital stay (Figure 2).

One week later, he was discharged with instructions for treatment with levetiracetam and triflusal for his CAD. During his follow-up one year after the episode as an outpatient in our clinic, he had a complete recovery without any other reported episodes of bleeding diathesis. Clotting assays remain between normal ranges. Written informed consent was obtained from the patient.

## DISCUSSION

Acquired coagulation factor inhibitors are a rare group of coagulation disorders. Autoimmune mediated responses lead to the emergence of autoantibodies that act as coagulation factor inhibitors. The latter lead to the depletion or deactivation of specific coagulation factors (1). Factor VIII is the most commonly affected coagulation factor, but inhibition may be directed against any factor (1,4,5).

Acquired factor V inhibitor is a rare form of factor V deficiency. The disease ranges from completely asymptomatic crossed upon routine laboratory work to major bleeding events such as intracranial haemorrhage or retroperitoneal bleeding (2,5). As in most cases, acquired coagulation inhibitors have been correlated with surgery, malignancies, drugs, previous blood transfusions and autoimmune disorders (6). Over 50% of the reported cases were described in surgical patients that received treatment with bovine thrombin (7). Bovine thrombin may contain bovine factor V that could be a strong immunological stimulus for the development of antibodies against factor V. After the substitution of bovine thrombin with human thrombin, most factor V inhibition cases were associated with antibiotics and malignancies (8,9).

Our patient underwent a major cardiothoracic surgery but bovine thrombin was not used. The diagnosis was established after the failure to reverse the PT and APTT prolongation with 50:50 mixing studies and the actual detection of the inhibitor with the Bethesda method at a very high concentration (64 BU). The emergence of the inhibitor could be attributed to the recent surgery and to the treatment with ceftazidime, a third generation cephalosporin (9).

Acquired factor VIII inhibition (acquired haemophilia A-AHA) is the most common disorder that is reflected in previous attempts to establish treatment guidelines (1,2). Due to the scarcity of cases of acquired factor V inhibition, there is limited evidence to support strong therapeutic recommendations. Treatment options are similar to those of acquired haemophilia A and include the following approaches: 1) control of bleeding, mostly achieved using a bypassing agent such as recombinant factor VIIa or activated prothrombin complex concentrate, even though FFP and PLT transfusion can be used; 2) eradicate the inhibitor by introducing immunosuppression with steroids and cytotoxic agents (recently anti-CD20 monoclonal antibodies have been used with encouraging results, especially in cases of acquired haemophilia A and; 3) the use of IV immunoglobulin, which remains controversial (2,5,6,9). The point of therapeutic regimen induction remains elusive.

Our patient was initially transfused upon admission with FFP and was started immediately on corticosteroids in combination with IV IGs as literature suggests. An immunosuppression regimen was escalated with the addition of rituximab and cyclophosphamide. Rituximab has slowly found its place amongst treatment regimens of acquired haemophilia A and other coagulation inhibitors, including that of factor V. Therefore, rituximab, alone or in combination with immunosuppressive drugs, is the main component of second-line treatment in case of a lack of response to first-line treatment within 8-12 weeks (grade 2B recommendation) (6,10,11). Bypass therapy with rFVIIa was introduced when the patient presented with seizures and a CT scan revealed a larger intracerebral haemorrhage.

After immunotherapy, the inhibitor levels were not repeated, mainly due to the fact that coagulation screening results were between normal ranges showing no indication of the presence of the inhibitor and increased cost.

In conclusion, more research is needed in the field of coagulation factor inhibitors. More and more conditions and drugs are correlated with the condition. Physicians should be alert in order to quickly identify the disease so that patients’ treatment will be immediate and targeted.

## Figures and Tables

**Table 1:**
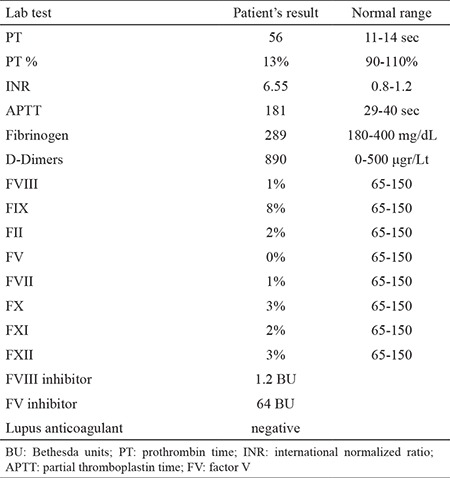
Patient’s coagulation tests during admission. Levels of Coagulation Factors and detection of FV inhibitor (64 BU)
